# Daily mean temperature and HFMD: risk assessment and attributable fraction identification in Ningbo China

**DOI:** 10.1038/s41370-021-00291-y

**Published:** 2021-02-05

**Authors:** Rui Zhang, Zhehan Lin, Zhen Guo, Zhaorui Chang, Ran Niu, Yu Wang, Songwang Wang, Yonghong Li

**Affiliations:** 1 Chinese Center for Disease Control and Prevention, Beijing, 102206 China; 2 China Population Communication Center, Beijing, 100013 China; 3 Institute of Medical Information/Medical Library, CAMS & PUMC, Beijing, 100020 China; 4 National Institute for Nutrition and Health, Chinese Center for Disease Control and Prevention, Beijing, 100050 China; 5 National Institute of Environmental Health, Chinese Center for Disease Control and Prevention, Beijing, 100021 China

**Keywords:** Hand, foot, and mouth disease (HFMD); Meteorological factors; Temperature; Distributed lag non-linear model (DLNM); Ningbo

## Abstract

**Background:**

Hand, foot, and mouth disease (HFMD) remains a significant public health issue, especially in developing countries. Many studies have reported the association between environmental temperature and HFMD. However, the results are highly heterogeneous in different regions. In addition, there are few studies on the attributable risk of HFMD due to temperature.

**Objectives:**

The study aimed to assess the association between temperature and HFMD incidence and to evaluate the attributable burden of HFMD due to temperature in Ningbo China.

**Methods:**

The research used daily incidence of HFMD from 2014 to 2017 and distributed lag non-linear model (DLNM) to investigate the effects of daily mean temperature (Tmean) on HFMD incidence from lag 0 to 30 days, after controlling potential confounders. The lag effects and cumulative relative risk (CRR) were analyzed. Attributable fraction (AF) of HFMD incidence due to temperature was calculated. Stratified analysis by gender and age were also conducted.

**Results:**

The significant associations between Tmean and HFMD incidence were observed in Ningbo for lag 0–30. Two peaks were observed at both low (5–11 °C) and high (16–29 °C) temperature scales. For low temperature scale, the highest CRR was 2.22 (95% CI: 1.61–3.07) at 7 °C on lag 0–30. For high temperature scale, the highest CRR was 3.54 (95% CI: 2.58–4.88) at 24 °C on lag 0–30. The AF due to low and high temperature was 5.23% (95% CI: 3.10–7.14%) and 39.55% (95% CI: 30.91–45.51%), respectively. There was no significant difference between gender- and age-specific AFs, even though the school-age and female children had slightly higher AF values.

**Conclusions:**

The result indicates that both high and low temperatures were associated with daily incidence of HFMD, and more burdens were caused by heat in Ningbo.

## Introduction

Hand, foot, and mouth disease (HFMD) is a common infectious disease in China. It was first reported in New Zealand in 1957 along with the isolation of Coxsackie virus as the pathogen [1]. This emerging infectious disease was then named as HFMD according to its typical symptoms in 1959 [2]. Infants and children under 6 years old are more likely to get this disease, though elder children and adults could also be infected. HFMD can be caused by various enteroviruses, including Coxsackie virus A16 and Enterovirus 71. Main clinical symptoms of HFMD include fever, mouth ulcers, and vesicles on the hands and feet. In most cases, the disease is mild and self-limiting. But a small proportion can rapidly develop neurological and systemic complications, including brainstem encephalitis and poliomyelitis-like acute flaccid paralysis [3].

Previous studies have shed light on the effect of meteorological factors such as temperature, relative humidity, and altitude on HFMD [4–9]. However, many studies have shown that the association between the incidence of HFMD and seasons or temperatures varied with different regions. Some areas have the peak once a year, such as in summer in Japan [10, 11], and in autumn in Finland [12]. Two peaks may occur in other regions, such as in summer and autumn in the United Kingdom [13, 14] and Belgium [15]. The incidence trends of HFMD are different in North and South China. In northern China, HFMD outbreaks in June among cities, such as Beijing [16, 17] and Hefei [8]. In several cities of southern China, like Kunming [18] and Guangdong [19, 20], the HFMD outbreaks in May and October–November. These discrepancies could arise from various local climatic conditions, differences in socioeconomic status, and the demographic characteristics of different regions. Our understanding of the impact of seasonal and meteorological variables on disease transmission remains limited. Further research is required to study the effects of meteorological variables on the incidence of HFMD.

At present, attributable risk has been used to study the quantitative dependence and regularity between risk factors and morbidity and mortality of infectious diseases. It has transformed factors such as lifestyle, physical, meteorological, and pollution indicators into measurable indicators for predicting the risk of disease or death in the future. An understanding of the temperature contributing to disease burden is critical for determining research priorities and informing national health policy. However, the contribution of temperature to the incidence of HFMD burden has not been quantified in Ningbo.

Therefore, this research aims to analyze the association between daily mean temperature and incidence of HFMD in Ningbo, as well as evaluate the attributable fraction (AF) of HFMD due to temperature. Thus, reliable basis for the prevention and control of HFMD in Ningbo could be provided.

## Materials and methods

### Geography

Ningbo locates in the Southern China and ranges in latitude from 28°51′ to 30°33′N and in longitude from 120°55′ to 122°16′E. It bounds on the east by the East China Sea and Zhoushan Archipelago, on the north by Hangzhou Bay, across which it faces Jiaxing and Shanghai, on the west by Shaoxing, and on the south by Taizhou. Its land area is 9714 km^2^, while oceanic territory amounts to 9758 km^2^. At the end of 2017, the permanent resident population of Ningbo was 8.05 million.

### Climate

Ningbo has a humid subtropical climate with four distinctive seasons, characterized by hot, humid summers and chilly, cloudy and dry winters (with occasional snow). The mean annual temperature is 16.4 °C, with monthly daily averages ranging from 4.7 °C in January to 28.0 °C in July. The city receives an average annual rainfall of 1480 mm and is affected by the plum rains of the Asian monsoon in June, when average relative humidity also peaks.

### Data collection

The daily incidence data of HFMD in Ningbo from January 2014 to December 2017 were applied from the official website of The Data-center of China Public Health Science [21]. The diagnostic criteria of HFMD were based on the clinical criteria set by the Hand, Foot, and Mouth Disease Control and Prevention Guide published by the National Health Commission of the People’s Republic of China [22]. To identify vulnerable populations, the data was reclassified by gender (male, female) and age (0–3, 4–5, ≥6 years old).

The daily mean temperature (°C), daily mean relative humidity (%) and daily precipitation (mm) were observed at Yinzhou station (29°47′N, 121°33′E), the national meteorological monitoring station in the central city of Ningbo. These data were provided by the China Meteorological Administration.

### Methods

A distributed lag non-linear model (DLNM) with quasi-Poisson regression was applied to evaluate the association between daily mean temperature and incidence of HFMD in Ningbo. The DLNM model was defined by the following formula [23]:

$$\log \left[ {E\left( {Y_t} \right)} \right] =	 \;\alpha + cb\left( {Tmean_t,\,lag = 30} \right) + ns\left( {time_i,df = 7/year} \right) \\ 	 + ns\left( {RHmean,3} \right) + \beta DOW_t$$

where *Y*_*t*_ represents the expected number of HFMD incidence on day *t*. *α* is the intercept. *Tmean*_*t*_ is the daily mean temperature on day *t*. *cb* refers to the crossbasis function, which specifies the exposure-lag-response relationship simultaneously in the exposure-response and lag-response dimensions; a quadratic B spline (*bs*) with 4 degrees of freedom (*df*) was used for the exposure-response relationship and natural cubic splines (*ns*) with 4 degrees of freedom for the lag-response relationship. The lag day up to 30 days reflects the maximum lag day of the temperature effect. A smooth function of time with 7 degrees of freedom per year was used in the model to control the seasonality and long-term trends. Daily mean relative humidity (*RHmean*), as potential confounding variables, was modeled as a natural cubic spline (*ns*) with 3 degrees of freedom. *DOW* stands for day of the week, which was entered as a categorical variable, and *β* is the coefficient of *DOW* [23–26].

The reference temperature for the DLNM analysis was identified according to the relationship between daily mean temperature and incidence of HFMD, that is, the temperature, at which the incidence of HFMD had the relative lower risk was identified as the reference temperature for this study. Cumulative relative risks (CRRs) for specific temperature scales under certain lag days were calculated to assess the effects of temperature on the incidence of HFMD.

AF was calculated to evaluate the incidence burden of HFMD caused by temperature. There are two ways to define AF within the dlnm framework. One is backward perspective, the other is forward perspective. Backward perspective is a commonly used explanation of lag effect in research, but its calculation process is more complicated than forward perspective. The principle and calculation of forward perspective are relatively simple, but the actual risk may be underestimated [26]. In this study, we used backward perspective to calculate AF. The backward AF *b*-*AF*_*x,t*_ at time *t* were obtained by the following formulas [26]:

$$b - AF_{x,t} = 1 - exp\left( { - \mathop {\sum}\nolimits_{l = l_0}^L {\beta _{x_{t - l},l}} } \right)$$

*l*_0_ and *L* correspond to minimum and maximum lags, respectively.

Finally, we calculated the empirical confidence interval values through Monte Carlo simulations [27] and the related 2.5th and 97.5th percentiles of multivariate normal distribution were interpreted as 95% empirical confidence intervals [26].

Stratified analyses by gender and age were also conducted. The statistical significance tests of gender- and age-specific differences were conducted by using the formula below [28]:

$$\left( {\widehat {Q_1} - \widehat {Q_2}} \right) \pm 1.96\sqrt {S\widehat {E_1}^2 + S\widehat {E_2}^2}$$

where $\widehat {{\mathrm{Q}}_1}\,{\mathrm{and}}\,\widehat {{\mathrm{Q}}_2}$ are the estimates for the two categories, and ${\mathrm{S}}\widehat {{\mathrm{E}}_1}$ and ${\mathrm{S}}\widehat {{\mathrm{E}}_2}$ are their respective standard errors.

### Sensitivity analysis

We conducted the collinear diagnosis to see if there is collinearity among daily mean relative humidity (RHmean) and daily precipitation. Sensitivity analysis was performed by changing the df of RHmean, adjusting and without adjusting RHmean and daily precipitation, respectively.

Data analysis was conducted using R software 3.6.2 with the package ‘dlnm’ [23]. The ‘dlnm’ package was used for fitting DLNM. For all statistical tests, statistical significance as a two-tailed *P* < 0.05.

## Results

### Descriptive analysis

Descriptive statistics for the number of HFMD cases based on gender and age, as well as meteorological variables including daily mean temperature, daily mean relative humidity, and daily precipitation are summarized in Table 1. A total of 129,897 HFMD cases from January 2014 to December 2017 were included in our analyses, of which 76,846 (59.16%) were male and 53,051 (40.84%) were female. The group aged 0–3 years, 4–5 years, and aged ≥6 years accounted for 76.43%, 17.19%, and 6.38%, respectively. The mean value of daily mean temperature was 17.46 °C, the standard deviation was 8.42 °C, the minimum value was −4.5 °C, and the maximum value was 32.9 °C. The mean value of daily mean relative humidity was 79.75%, the standard deviation was 11.18%, the minimum value was 34%, and the maximum value was 100%. The mean value of daily precipitation was 5.0 mm, the standard deviation was 14.38 mm, the minimum value was 0 mm, and the maximum value was 276.2 mm.

**Table 1:** Descriptive statistics of daily incidence of HFMD and meteorological factors in Ningbo, 2014–2017.

	Total	Mean ± SD	Min	Max	Proportion
Daily mean incidence (cases)	129,897	88.91 ± 76.84	1	479	–
Sex					
Male	76,846	52.6 ± 45.76	0	302	59.16%
Female	53,051	36.31 ± 31.71	0	183	40.84%
Age					
0–3	99,284	67.96 ± 56.29	1	309	76.43%
4–5	22,331	15.28 ± 16.83	0	124	17.19%
≥6	8282	5.67 ± 6.13	0	46	6.38%
Daily mean temperature (°C)	–	17.46 ± 8.42	−4.5	32.9	–
Daily mean relative humidity (%)	–	79.75 ± 11.18	34	100	–
Daily precipitation (mm)	–	5.0 ± 14.38	0	276.2	–

**Fig. 1:**
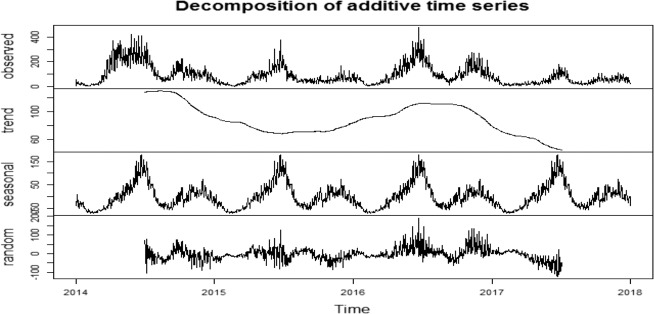
Decomposition of additive time series of daily incidence of HFMD in Ningbo (from January 2014 to December 2017).

Figure 1 shows the decomposition analysis of additive time series of daily incidence of HFMD in Ningbo from January 2014 to December 2017, which included the trend of the observed cases, the long-term and seasonal trends and the random variation. The long-term trend showed that the overall incidence of HFMD in Ningbo presented a downward trend from 2014 to 2015, followed by slowly rising in 2016 and then decreasing in 2017. The seasonal lines showed a strong seasonality that a bimodal seasonal pattern was observed in Ningbo, which was characterized by peaks in HFMD incidence in the summer (June) and early winter (November). And the random line showed the randomness of the data. It was indicated that the dataset of the incidence of HFMD during 2014–2017 in Ningbo was a typical time series and the meteorological variables might be associated with HFMD.

### Associations between temperature and HFMD

**Fig. 2:**
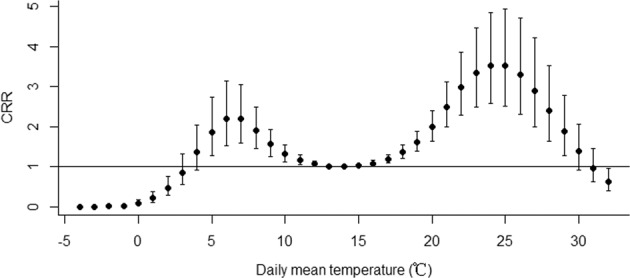
Cumulative relative risk (CRR) of daily incidence of HFMD associated with daily mean temperature (on lag 0–30 days and reference temperature of 14 °C).

Figure 2 shows the CRR of daily HFMD incidence associated with daily mean temperature on lag 0–30. Analysis of the crude relationship revealed that the potential overall CRR of HFMD had an approximately M-shape with two peaks during 5–11 °C and 16–29 °C which was consistent with the bimodal seasonal trend. It was demonstrated in Fig. 2 that the lowest point between the two peaks was 14 °C, so we chose 14 °C as the reference temperature. The highest risk was at 7 °C during 5–11 °C and at 24 °C during 16–29 °C, so 7 and 24 °C were chosen as the representative low and high temperatures to calculate relative risk (RR) and CRR, respectively.

**Fig. 3:**
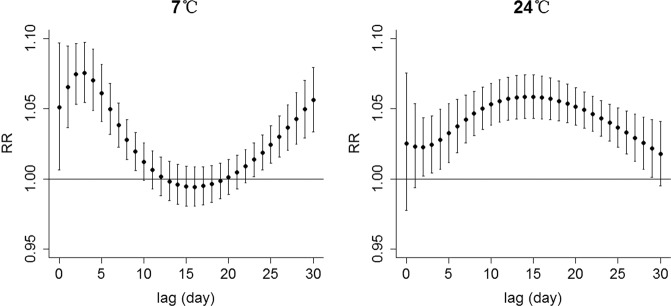
Relative risk of daily incidence of HFMD associated with low and high temperatures (on lag 0–30 days and reference temperature of 14 °C).

Figure 3 shows the RR of daily incidence of HFMD associated with high and low temperatures. It indicated that the temperature-HFMD association was non-linear, immediate, and lasted throughout 30 days lag periods. The RR of daily incidence of HFMD at 7 °C were more prominent over shorter 10 days lag periods with the highest RR of 1.08 (95% CI: 1.06–1.10) on lag 3 (Table 2) and diminished over time, whereas the effects at 24 °C were persistent over longer 30 days lag periods with the highest RR of 1.06 (95% CI: 1.04–1.07) on lag 14 (Table 2).

**Table 2:** Relative risks and cumulative relative risks of incidence of HFMD associated with low and high temperatures (reference temperature: 14 °C).

	RR (95% CI)		CRR (95% CI)	
	7 °C, lag 3	24 °C, lag 14	7 °C, lag 0–30	24 °C, lag 0–30
Overall	1.08 (1.06, 1.10)	1.06 (1.04, 1.07)	2.22 (1.61, 3.07)	3.54 (2.58, 4.88)
Sex				
Male	1.08 (1.06, 1.11)	1.05 (1.03, 1.07)	2.50 (1.77, 3.55)	3.12 (2.22, 4.40)
Female	1.07 (1.04, 1.09)	1.07 (1.05, 1.09)	1.89 (1.31, 2.71)	4.23 (2.94, 6.07)
Age				
0–3	1.07 (1.05, 1.09)	1.05 (1.04, 1.07)	2.04 (1.47, 2.84)	3.31 (2.40,4.59)
4–5	1.11 (1.07, 1.14)	1.08 (1.06, 1.11)	3.64 (2.19, 6.03)	5.08 (3.08, 8.36)
≥6	1.07 (1.03, 1.12)	1.07 (1.04, 1.10)	1.84 (0.93, 3.63)	3.88 (2.00, 7.55)

It was shown in Table 2 that the CRR at 7 °C from lag 0–30 was 2.22 (95% CI: 1.61–3.07) and the CRR at 24 °C from lag 0–30 was 3.54 (95% CI: 2.58–4.88). The results displayed that both gender- and age-specific difference were statistically insignificant. At the low temperature of 7 °C, the CRR of male was 2.50 (95% CI: 1.77–3.55) and that of female was 1.89 (95% CI: 1.31–2.71). The CRR of group aged 0–3 years, 4–5 years, and ≥6 years was 2.04 (95% CI: 1.47–2.84), 3.64 (95% CI: 2.19–6.03), and 1.84 (95% CI: 0.93–3.63), respectively. At the high temperature of 24 °C, the CRR of male was 3.12 (95% CI: 2.22–4.40), and that of female was 4.23 (95% CI: 2.94–6.07). The CRR of group aged 0–3 years, 4–5 years, and ≥6 years was 3.31 (95% CI: 2.40–4.59), 5.08 (95% CI: 3.08–8.36), and 3.88 (95% CI: 2.00–7.55), respectively.

### Attributable fraction of HFMD due to temperature

Table 3 presents the AF of HFMD due to temperature for the overall group as well as gender- and age-specific subgroups. It was shown that the AFs were higher for hot temperatures (16–29 °C) than those for cold temperatures (5–11 °C). The estimated overall AF due to temperature was 45.50% (95% CI: 36.21–52.48%). The AF due to cold temperatures (5–11 °C) was 5.23% (95% CI: 3.10–7.14%) and the AF due to hot temperatures (16–29 °C) was 39.55% (95% CI: 30.91–45.51%). The AF of male and female due to temperature was 42.98% (95% CI: 31.51–51.23%) and 48.26% (95% CI: 39.09–54.77%), respectively. The group aged 4–5 years (AF: 53.34%, 95% CI: 40.91–60.96%) and ≥6 years (AF: 50.59%, 95% CI: 26.93–62.89%) seemed to have higher AF values than the group aged 0–3 years (AF: 43.44%, 95% CI: 34.14–51.41%), but there was no significant difference. The similar trends of the age-specific AFs due to cold and hot temperatures were also observed.

**Table 3:** Attributable fraction of incidence of HFMD due to temperature (AF 95%CI, %).

Group	Overall	5–11 °C	16–29 °C
Overall	45.50 (36.21, 52.48)	5.23 (3.10, 7.14)	39.55 (30.91, 45.51)
Sex			
Male	42.98 (31.51, 51.23)	5.92 (3.60, 7.79)	36.61 (27.02, 44.29)
Female	48.26 (39.09, 54.77)	4.20 (1.55, 6.47)	43.15 (34.21, 48.94)
Age			
0–3	43.44 (33.14, 51.41)	4.67 (2.41, 6.59)	38.31 (29.88, 44.79)
4–5	53.34 (40.91, 60.96)	8.36 (5.25, 10.81)	44.16 (34.93, 51.28)
≥6	50.59 (26.93, 62.89)	3.99 (−1.79, 7.50)	43.74 (26.05, 56.20)

### Sensitivity analysis

The result of collinear diagnosis showed that there was no serious collinearity among RHmean and daily precipitation (correlation coefficient is 0.35). The sensitivity analysis results (see Table S1) showed that the CRRs remained unchanged, which indicated that the models were stable.

## Discussion

We assessed the association between daily mean temperature and daily incidence of HFMD in Ningbo and two peaks were discovered during specific cold (5–11 °C) and hot (16–29 °C) temperatures. What is more, to our best knowledge, for the first time we evaluated the AF of HFMD incidence due to temperature in Ningbo and found that much more disease burden of HFMD could be attributed to high temperature than that attributed to cold temperature. Stratified analysis implied that the occurrence of HFMD of school-age children seemed to be more sensitive to temperature than the group aged 0–3 years.

In this study, we found that the incidence of HFMD in Ningbo has semiannual peaks, including a higher peak in summer and the lower one in early winter. The result is consistent with the previous studies which demonstrated that HFMD outbreaks once a year in high latitudes regions but twice a year in tropical and subtropical regions [2, 10, 19].

Several previous studies have shed light on the effect of meteorological factors such as temperature on HFMD [4–9, 19, 29]. However, the results have shown that the association between the incidence of HFMD and temperature varied with different regions [24, 29–34]. Chen et al. [35] found that the occurrence of HFMD epidemics was bimodal in Wuhan, one peak occurred when the monthly average temperature was below 15 °C during autumn-winter and the other occurred when the monthly average temperature exceeds 25 °C in summer. The study in Guangzhou [19] also found that the association between the daily incidence of HFMD and the daily mean temperature increased rapidly when the daily mean temperature was below 25 °C but flattened above 25 °C. While our result showed that the risk of HFMD increased during both low temperature from 5 to 11 °C and high temperatures from 16 to 29 °C of daily mean temperatures, but declined at both extremely cold (<5 °C) and hot (>29 °C) temperatures. The inconsistency of these findings can be partly attributable to the diversity of methodologies and data sources, but it also implies that the temperature-HFMD relationship might be modified by some location-specific variables. The heterogeneity across studies is still poorly understood which hinder a more comprehensive characterization of the temperature-HFMD relationship.

As some previous studies suggested, our results imply that the effect of temperature on HFMD may involve with complex mechanisms. Some evidential studies found that temperature could affect the breeding, growth, and transmission of pathogens, as well as human behavior [36]. First, HFMD can be transmitted either by exposure to infectious individuals or contaminated environment. During the suitable temperatures (such as 5–11 °C and 16–29 °C), individuals are more likely to have outdoor activities, thereby suitable temperature may increase the chances that susceptible individuals contact with infectious individuals or contaminated environment. Conversely, people will reduce outdoor activities and take some protective measures at extreme temperature. Second, both extremely cold and hot temperatures can reduce the reproduction of pathogens. Temperature is considered as the main factor determining enterovirus inactivation in the environment [37]. According to the previous studies in the laboratory, EV-A71 and CVA16 are lack of a thermostatic mechanism, and their reproduction and survival rates are strongly affected by fluctuations in temperature. The EV-A71 survives at 20 °C slightly better than at 25 °C [19], and at 36 °C is more active than at 39 °C [38]. Therefore, extremely temperature may shorten the survival time of enterovirus in the environment. All of above might explain the two peaks during specific suitable temperature scales and the dropping down of risk at extreme temperature.

Measures of attributable risk are an integral part of epidemiological analyses, particularly when aimed at the planning and evaluation of public health interventions. It was presented in our results that the overall AF of HFMD incidence due to hot temperatures (39.55%) was significantly higher than that due to cold temperatures (5.23%). This results indicated that when the temperature is between 16 and 29 °C in summer in Ningbo, public-health authorities should prepare fully to respond to a possible epidemic of HFMD, including increasing access to health-care resources, the distribution of scientific knowledge to the public, medical staff and public health personnel, the availability of essential medical equipment, active disease surveillance, and the design of other more specific control measures to mitigate the risk of disease transmission.

Although difference of temperature effects between males and females were not statistically significant, the daily incidence of HFMD tended to be higher in males than in females. This result is consistent with other studies [2, 19]. One possible reason is that boys may be more active and thus have more chances to infect the disease.

It was displayed in our study that the number of the HFMD cases of group aged 0–3 years was the highest. However, the AFs of group aged 4–5 years and ≥6 years were both higher than the AF of group aged 0–3 years, which meant that the occurrence of HFMD of the school-age children be more susceptible to the ambient temperature. Our results indicated that more attention should also be paid to the school-age children especially by strengthening the hand hygiene and the monitor of symptoms and diseases in kindergarten and primary school especially during periods with specific ambient temperature.

Some limitations of this paper should be acknowledged. First, we used ambient temperature data monitored at a fixed location as most previous epidemiological studies, rather than individual real exposure data, which could lead to measurement errors for exposure. Second, as the availability of the data, we did not control the effect of air pollution on the incidence of HFMD. Third, our study focused on Ningbo, a southern city in China. The findings need to be verified in more other cities or regions. However, our results warrant further research on climate change and the incidence of HFMD and provide scientific evidence for the planning of control and prevention of HFMD in Ningbo.

## Conclusions

Both high and low temperatures were associated with daily incidence of HFMD, and more burdens were caused by heat in Ningbo. The results would provide scientific evidence to the threshold setting of HFMD early warning system and help local health departments to provide early warnings to control the risk before reaching its peak and implement effective interventions to reduce the burden of HFMD due to temperature.

## Supplementary information

Supplementary TableS1
