# Supplementary material for: Daily mean temperature and HFMD: risk assessment and attributable fraction identification in Ningbo China
Source: J Expo Sci Environ Epidemiol. 2021 Feb 5;31(4):664–71. doi: 10.1038/s41370-021-00291-y (PMC8263339; doi:10.1038/s41370-021-00291-y)
Supplement: Supplementary file 1 — Supplementary TableS1 [file 41370_2021_291_MOESM1_ESM.docx]

**Table S1. CRR of HFMD incidence by changing the df of RHmean and adjusting and without adjusting RHmean and daily precipitation**

|  | **7℃, lag 0-30** | **24℃, lag 0-30** |
| --- | --- | --- |
| Adjusting RHmean^#^ | |  |
| df=3 | 2.22(1.61, 3.07) | 3.54(2.58, 4.88) |
| Changing the df of RHmean | | |
| df=2 | 2.19(1.59, 3.03) | 3.51(2.55, 4.82) |
| df=4 | 2.23(1.62, 3.08) | 3.47(2.52, 4.78) |
| Without adjusting RHmean | 2.09(1.52, 2.88) | 3.61(2.62, 4.96) |
| Adjusting daily precipitation | | |
| df=3 | 2.06(1.49, 2.84) | 3.61(2.63, 4.97) |

**CRR means cumulative relative risks, RHmean means daily mean relative humidity, df means degree of freedom.*

*^#^: Without adjusting daily precipitation*
